# Alprazolam addiction: The case study

**DOI:** 10.1192/j.eurpsy.2021.1724

**Published:** 2021-08-13

**Authors:** O. Pityk, N. Karbovskyi, N. Galyga, O. Rubanets

**Affiliations:** 1 Psychiatry, Narcology And Medical Psychology Department, Ivano-Frankivsk National Medical University, Ivano-Frankivsk, Ukraine; 2 Student, Ivano-Frankivsk National Medical University, Ivano-Frankivsk, Ukraine; 3 Neuroses And Borderline Disorders Department, Precarpathian Regional Clinical Center of Ivano-Frankivsk Regional Council, Ivano-Frankivsk, Ukraine; 4 Outpatient Department, Precarpathian Regional Clinical Center of Ivano-Frankivsk Regional Council, Ivano-Frankivsk, Ukraine

**Keywords:** Alprazolam, Addiction, Psychoeducation

## Abstract

**Introduction:**

Alprazolam is an anxiolytic, a benzodiazepine derivative of the middle duration of action. It is one of the most frequently prescribed medication for the treatment of anxiety and panic disorder. Under the action of a drug, a person feels incredible ease, a sense of euphoria, absence of problems, a sense of safety.

**Objectives:**

A 55-year woman was admitted to psychiatric clinic in Ivano - Frankivsk.

**Methods:**

She was assesed by the clinicopsychopatological method (clinical interview) and additional methods (MRI, EEG, pathopsycological assessment).

**Results:**

The main findings were: atactic procession, tremor of the limbs and the whole body, poor attention, speech impairment, retarded thinking, fixation and reproductive amnesia with the components of progressive amnesia, change handwriting. The mood is lowered with unstable affect, lack of insight. She reported burning and tingling of the head as a main problem. She developed amotivation, bad activity and drowsiness, bradycardia, decreased blood pressure. She took Alprazolam during a period of 1,5 year in gradually increasing doses. The last dose was 12 tablets of Alprazolam per day. The patient was consulted again in a year. She does not take Alprazolam. She takes valproate and escitalopram. She did not demonstrate severe neurological symptoms which were seen a year ago.

**Conclusions:**

Thus, though alprazolam is one of the best anxiolytic substance it should be prescribed only by the doctor for a short course (no more than 4-5 weeks). The treatment must include psychoeducation in order to make patients be aware about possible addiction and unsafety of prolonged and uncontrolled usage of alprazolam.

**Disclosure:**

No significant relationships.

